# Attribute Preferences for Somatostatin Analogues in Neuroendocrine Tumours (NETs) Among Patients, Clinicians and Nurses in Australia

**DOI:** 10.1002/cam4.71323

**Published:** 2025-11-12

**Authors:** Simon J. Fifer, Karen Winkler, Meredith Cummins, Hima Cherian

**Affiliations:** ^1^ Community and Patient Preference Research (CaPPRe) Sydney New South Wales Australia; ^2^ NeuroEndocrine Cancer Australia Blairgowrie Victoria Australia; ^3^ Ipsen Australia South Yarra Victoria Australia

**Keywords:** discrete choice experiment, long‐acting somatostatin analogues (LA SSAs), neuroendocrine tumours (NETs), patient preferences, treatment preferences

## Abstract

**Background:**

Long‐acting somatostatin analogues (LA SSAs) are commonly used as first‐line treatment in well‐differentiated low and intermediate grade 1–2 gastroenteropancreatic neuroendocrine tumours (GEP‐NETs). As a long‐term treatment option for NETs, treatment preferences should be taken into consideration. The objective of this study is to evaluate the relative importance of different LA SSA treatment features among patients with NETs, clinicians and nurses in Australia.

**Methods:**

A discrete choice experiment (DCE) method was used to analyse treatment preferences for NETs, embedded in an online survey. Participants could choose between three hypothetical treatment options: ‘deep subcutaneous injectable treatment’, ‘deep intramuscular injectable treatment’, ‘oral treatment’; and an opt‐out. Each option was described by seven attributes: ‘Progression free survival’ (PFS), ‘symptom control for diarrhoea and flushing’, ‘risk of gastrointestinal (GI) side effects’, ‘frequency of administration’, ‘treatment administration,’ ‘treatment delivery,’ and ‘availability of patient support’. A Mixed Multinomial Logit model (MMNL) was used for analysis.

**Results:**

A total of 54 patients with NETs, 27 clinicians, and 9 nurses completed the DCE (of 33, 26 and 20 planned respectively). The statistical model showed an overall preference for oral administration. ‘PFS’ was the most important attribute, followed by ‘symptom control for diarrhoea and flushing’ and ‘risk of GI side effects’. ‘PFS’ and ‘symptom control for diarrhoea and flushing’ were also found to be more important to healthcare professionals (HCPs, i.e., clinicians and nurses) than patients with NETs, while the attributes ‘symptom control for diarrhoea and flushing’ and ‘risk of GI side effects’ were found to be more important in non‐metropolitan areas (vs. metropolitan areas).

**Conclusions:**

Analysis showed how patients with NETs and HCPs prioritise treatment attributes for NETs differently. Because the duration of use of LA SSA for the treatment of NETs is often long, these differences highlight the importance of involving the patient when making treatment decisions.

**Abbreviations**
DCE: discrete choice experiment
GI: gastrointestinal
HCPs: healthcare professionals
IM: intramuscular
LA: long‐acting
MMNL: mixed multinomial logit model
MNL: multinomial logit model
NETs: neuroendocrine tumours
PFS: progression free survival
RUT: random utility theory
SC: subcutaneous
SE: standard errors
SSAs: somatostatin analogues

## 1 Background

Neuroendocrine tumours (NETs) are a relatively rare condition in which neuroendocrine cells develop into tumours [1]. These tumours can be found throughout the body [2], but the gastrointestinal tract and the pancreas are among the most common locations [3]. The incidence of NETs varies across geographic regions [3] with an estimate of 5437 new cases in 2022 in Australia [4, 5]. The incidence of NETs in Australia and other countries such as the US has been increasing over time [4, 6]. Due to the slow growth of NETs and lack of awareness and understanding of vague symptoms, most patients with NETs are diagnosed at advanced stages [7].

The treatment of NETs is largely dependent on the functional status and the stage of the tumour [8]. For those patients with advanced NETs, a number of therapeutic options are available which include somatostatin receptor targeting options [8]. Somatostatin analogues are recommended as a first‐line treatment in SST‐positive, advanced well‐differentiated low and intermediate grade 1–2 GEP‐NETs, both for their antisecretory and antiproliferative effects [7, 9]. Due to the long duration of use in patients with NETs [10, 11] preferences for SSA treatment attributes should be taken into consideration.

People's preferences for treatment are increasingly considered integral to the evaluation of new treatment options [12, 13, 14, 15] and to the treatment and care process [16, 17]. However, there is a paucity of research examining the treatment preferences of patients with NETs and those involved in their treatment and care. Discrete choice experiments (DCEs) are increasingly used to quantify patient and other stakeholder preferences for treatments in a broad range of diseases [18, 19, 20, 21] and allow for an understanding of the underlying characteristics, or attributes, of treatment that influence patient choice.

This is the first study to evaluate the relative importance of different LA SSA treatment features among patients with NETs as well as clinicians and nurses (collectively referred to as healthcare professionals (HCPs) from here on where applicable) in Australia, an important consideration in evaluating optimal treatment choice, care and management of patients diagnosed with NETs. A secondary objective of the study was to identify differences between subsegments of patients and HCPs where possible.

While conducted in Australia, these findings can be contextualised within broader international experience. Prior European and US studies have specifically examined SSA use in NETs, including real‐world treatment patterns and clinical outcomes [10, 11], as well as clinical guidelines recommending SSA therapy for NET management [8, 9].

Although non‐clinical in nature, (While this study focuses on patient preferences rather than clinical outcomes or biological mechanisms, it is important to recognise the broader therapeutic context. Recent research has advanced understanding of somatostatin analogues, including their mechanisms of action, long‐term efficacy, side‐effect profiles, and evolving use in combination or sequencing with other therapies. Acknowledging these developments helps situate patient‐centered evidence alongside clinical and biological considerations.), this study offers valuable insights that can inform clinical practice by capturing the preferences of patients and HCPs and understanding how they make trade offs between different aspects of care. The findings provide evidence to support more patient‐centred treatment decisions and help bridge the gap between clinical evidence and patient priorities, contributing to care that is better aligned with real‐world needs.

## 2 Methods

In a DCE, participants choose between different alternatives that vary in their attributes or features. By studying these choices, researchers can understand what factors are most important to people and how they make decisions. Although DCEs are used in many areas, they are especially helpful in healthcare research to explore complex decision‐making.

An online survey with a DCE component was designed to understand the treatment preferences of patients with NETs and HCPs caring for this type of patient. Such methods are recommended globally to assess the needs and experiences of patients with NETs and can be used to inform decision‐making by regulators and health technology assessors [22]. The survey was conducted between September 2021 and February 2022. While the DCE component was identical for patients with NETs and HCPs (with a slightly different framing dependent on respondent type, see details on study design below), the remainder of the survey varied somewhat in terms of background and demographic information collected from each respondent group. The DCE comprised a choice task in which participants were presented with different hypothetical treatment profiles and asked to select their preferred treatment.

### 2.1 Participants

A total of 54 patients with NETs, 27 clinicians and 9 nurses completed the survey (of 33, 26 and 20 planned, respectively). Participants were recruited through NeuroEndocrine Cancer Australia, specialist healthcare market research panels, and online research of clinic websites. Because NETs are a less common cancer and there is low awareness or knowledge in the general community as well as among HCPs, recruitment was challenging, especially for HCPs, resulting in a limited sample size. The eligibility criteria for the study are shown in Table 1. Participant eligibility was based on self‐reported information.

**TABLE 1:** Eligibility criteria.

Patients	Patients were required to be 18 years or older, citizens or permanent residents of Australia (to ensure eligibility for public health insurance, i.e., Medicare), and to have been diagnosed with NETs
Clinicians	Clinicians were required to specialise in oncology or endocrinology, have experience with caring for patients with NETs for at least 12 months, and to have seen at least one patient with NETs over the last 12 months
Nurses	Nurses were required to specialise in oncology or endocrinology, have experience with treating patients with NETs for at least 12 months, to have seen at least one patient with NETs over the last 12 months, and to have experience with administering LA SSA injections
All	Employees of a pharmaceutical company or a third party contracting for a pharmaceutical company were excluded from the study

### 2.2 Study Design

Qualitative interviews were conducted initially to obtain a greater awareness of the therapy area for NETs from the patient and HCP perspectives, especially regarding the use of and preferences for LA SSA injections.

A total of seven in‐depth interviews, each lasting between 40 and 60 min, were conducted on three patients with NETs, two clinicians and two nurses treating NETs. All interviews were conducted via telephone by an experienced researcher. Qualitative interviews provided a deeper understanding of the experiences and challenges in the treatment of NETs, as well as the attributes that contributed to treatment decision‐making and determined the appropriate terminology for each respondent group. The results of the qualitative research were critical to inform the design of the DCE.

The design of the DCE, including the selection of attributes (i.e., treatment characteristics) and levels for inclusion, was informed by existing literature, the qualitative in‐depth interviews, discussions with patient advocacy groups, and expert opinion. The following attributes were included in the DCE: ‘progression free survival’, ‘symptom control for diarrhoea and flushing’, ‘risk of gastrointestinal side effects’, ‘frequency of administration’, ‘treatment administration’, ‘treatment delivery’ and ‘availability of patient support’. Table 2 lists the seven treatment attributes and the levels associated as determined by the study team.

**TABLE 2:** DCE attributes, descriptions and levels.

Attribute	Description	Deep subcutaneous injectable treatment	Deep intramuscular injectable treatment	Oral treatment
Frequency of administration	How often the treatment needs to be injected/taken			Twice daily
				Once daily
		Every 2 weeks	Every 2 weeks	
		Every 4 weeks	Every 4 weeks	
		Every 6 weeks	Every 6 weeks	
		Every 8 weeks	Every 8 weeks	
Treatment administration	Who can administer treatment and where			Patient at home
		Self‐injection at home (either by patient or family member/carer)		
		Nurse/GP at patient's home	Nurse/GP at patient's home	
		Nurse/GP at GP practice	Nurse/GP at GP practice	
		Nurse/specialist at clinic/hospital	Nurse/specialist at clinic/hospital	
Treatment delivery	The format in which the treatment is delivered			Tablet
		Pre‐filled syringe	Pre‐filled syringe	
		Solution requires reconstitution (mixing) prior to administration	Solution requires reconstitution (mixing) prior to administration	
Progression free survival	The length of time during (and after) treatment that the disease is stable/under control, i.e., does not get worse	5 months	5 months	5 months
		15 months	15 months	15 months
		25 months	25 months	25 months
		35 months	35 months	35 months
		45 months	45 months	45 months
		55 months	55 months	55 months
Symptom control for diarrhoea and flushing	How well the treatment is able to control disease symptoms, i.e., flushing and diarrhoea, which may influence quality of life	90% of patients have control of symptoms	90% of patients have control of symptoms	90% of patients have control of symptoms
		75% of patients have control of symptoms	75% of patients have control of symptoms	75% of patients have control of symptoms
		60% of patients have control of symptoms	60% of patients have control of symptoms	60% of patients have control of symptoms
		45% of patients have control of symptoms	45% of patients have control of symptoms	45% of patients have control of symptoms
Risk of gastrointestinal (GI) side effects from treatment	GI side effects can include diarrhoea, abdominal pain, nausea, increase in blood glucose levels etc.	10% risk	10% risk	10% risk
		20% risk	20% risk	20% risk
		30% risk	30% risk	30% risk
		40% risk	40% risk	40% risk
Availability of patient support	Patient support provided by the manufacturer of the treatment helping with e.g., information materials or injection services	None	None	None
		Patient information materials (e.g., brochures, injection training video)		
		Financial support for diagnostic testing	Financial support for diagnostic testing	
		Exercise physiology and mental health counselling	Exercise physiology and mental health counselling	
		Nurse/GP training on how to self‐inject at patient's home		
		Nurse/GP injection service at patient's home	Nurse/GP injection service at patient's home	

**FIGURE 1:**
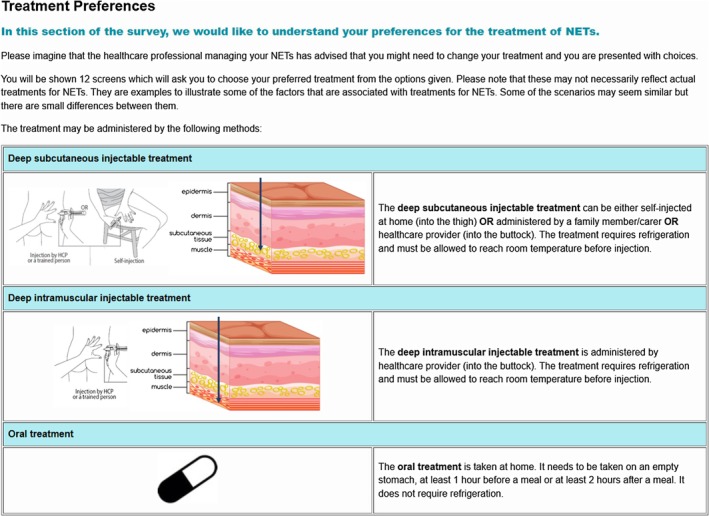
Framing of DCE choice task (example for patients).

A labelled design was used, where the hypothetical treatment profiles were displayed as ‘deep subcutaneous injectable treatment’, ‘deep intramuscular injectable treatment’ or ‘oral treatment’. To capture cases where respondents felt none of the presented treatments met their needs or preferences, an ‘opt‐out’ option was included. For patients with NETs currently on LA SSA treatment, the opt‐out was labelled ‘Remain on current treatment’, reflecting the possibility that they preferred their existing regimen over new options. For patients not on LA SSA treatment and for HCPs, the opt‐out was labelled ‘Neither of these treatments’, acknowledging that they may not view the presented options as suitable. The framing of the choice question was also slightly varied depending on the respondent type (see Figure 1 for an example for patients with NETs).

**FIGURE 2:**
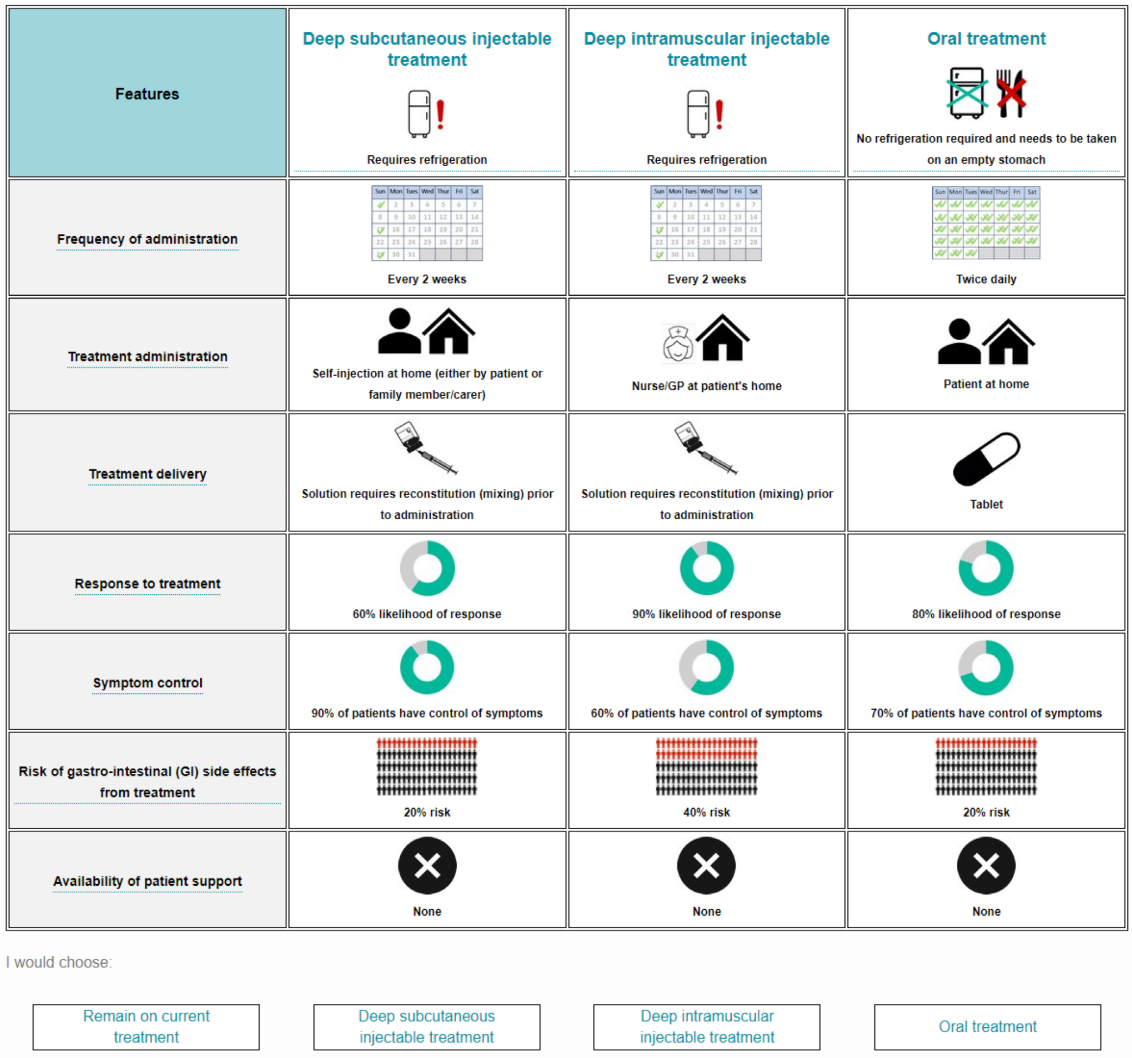
Example of a DCE choice task.

The experimental design followed good practice guidelines [23] and the combinations of levels presented in the tasks were designed using a D‐efficient design [24] in NGene. Naïve priors were used to account for the level direction in the design. A total of 72 scenarios were developed, and the final design included 12 choice scenarios per respondent in each of the six blocks, which were randomly distributed among the target sample. Each treatment profile displayed represented a different combination of the attribute levels displayed in Table 2. An example of a choice task displayed to patients with NETs is shown in Figure 2.

An initial pilot study phase was conducted to evaluate the content and design of the survey and DCE prior to use in the main study. Following the pilot, the range of levels for the ‘PFS’ attribute was reduced from 6–72 to 5–55 months prior to fully launching the study.

### 2.3 Sample Characteristics and Data Analysis Approach

Sixty‐five patients with NETs, 29 clinicians and 10 nurses in total completed the survey. Participants who finished the survey too quickly (< 7 min), gave nonsensical or inconsistent answers, or were suspected duplicates were excluded from the analysis. This resulted in the removal of 11 patients with NETs, two clinicians and one nurse from the sample.

The median time for survey completion was 39.4 min for patients with NETs, 19.0 min for clinicians and 22.1 min for nurses. Participants indicated that they had a good level of understanding of the task, with an average understanding of 9.2 for patients with NETs (SD = 1.1) on a 10‐point scale, 8.9 for clinicians (SD = 1.2) and 9.3 for nurses (SD = 1.0). Participants also found the task easy to complete, with an average ease rating of 7.6 (SD = 2.0) on a 10‐point scale for patients with NETs, 8.0 for clinicians (SD = 1.6) and 9.0 for nurses (SD = 1.3).

Descriptive statistics were used to summarise participants' demographic and disease/treatment background information (for patients with NETs) and treatment experience/practice details (for HCPs). Mean and standard deviation (SD) were reported for numeric variables, and percentages were reported for categorical variables. For the DCE analysis, the numerical attributes (frequency, progression free survival, symptom control, risk of GI side effects) were treated as continuous variables.

The theoretical basis for DCE is based on Random Utility Theory (RUT) initially developed by Thurstone [25] and further developed by McFadden [26], combined with Lancaster's theory of value [27]. Based on this theoretical framework, Louviere and Hensher [28] and Louviere and Woodworth [29] developed the empirical design approach to DCE.

A Mixed Multinomial Logit model (MMNL) was used to model DCE data in this study. This model accounts for preference heterogeneity (variation) by allowing marginal utility coefficients to be distributed randomly across respondents based on distributions [23, 30].

Data for patients with NETs and HCPs was pooled for overall model estimation and analysed for differences between patients (*n* = 54) vs. HCPs (*n* = 36), i.e., clinicians and nurses considered together. The data was also analysed for differences between respondents from metropolitan areas (respondents describing the area they live in as metropolitan or city areas) (*n* = 59) vs. non‐metropolitan areas (regional or rural areas) (*n* = 21). The model was specified and estimated in Nlogit version 6 (Econometric Software Inc.), and *p* < 0.05 criteria was used to determine statistical significance.

## 3 Results

### 3.1 Participant Demographics

Demographics for 54 patients with NETs, 27 clinicians and 9 nurses treating patients with NETs are displayed in Table 3. Most patients with NETs (74.1%) were receiving treatment with a LA SSA at the time of the study or were on ‘watch‐and‐wait’ (22.2%).

**TABLE 3:** Participant demographic characteristics.

Demographic characteristics		Patients with NETs (*N* = 54)	Clinicians (*N* = 27^a^)	Nurses (*N* = 9^a^)
Gender, *N* (%)	Male	19 (35.2)	16 (59.3)	3 (33.3)
	Female	35 (64.8)	10 (37.0)	6 (66.6)
	Prefer not to answer	—	1 (3.7)	—
Age, *N* (%)	18–30	1 (1.9)	—	—
	31–40	2 (3.7)	10 (37.0)	2 (22.2)
	41–50	5 (9.3)	10 (37.0)	5 (55.6)
	51–60	21 (38.9)	7 (25.9)	2 (22.2)
	61–70	16 (29.6)	—	—
	71–80	9 (16.7)	—	—
	81 or older	—	—	—
Location, *N* (%)	Metro/City	33 (61.1)	20 (74.1)	6 (66.7)
	Regional	16 (29.6)	5 (18.5)	3 (33.3)
	Rural	5 (9.3)	2 (7.4)	—
Currently on LA SSA treatment, *N* (%)	Yes	40 (74.1)		
	No	14 (25.9)		
Speciality, *N* (%)	Oncology		25 (92.6)	8 (88.9)
	Endocrinology		2 (7.4)	1 (11.1)
Years of experience treating NETs, *N* (%)	1–2 years		2 (7.4)	—
	3–4 years		5 (18.5)	1 (11.1)
	5–6 years		2 (7.4)	3 (33.3)
	7–8 years		6 (22.2)	1 (11.1)
	9–10 years		5 (18.5)	1 (11.1)
	More than 10 years		7 (25.9)	3 (33.3)

^a^ Please note small base size.

All but one patient in the study (98.1%) expressed a desire to be involved in the treatment decision at least to some extent, with the majority (63.0%) wanting to ‘make the treatment decision together with their doctor’ (see Table 4).

**TABLE 4:** Patient involvement in treatment decision.

Patient involvement in treatment decision *N* (%)	Patients with NETs (*N* = 54)
I prefer to make the treatment decision on my own	—
I prefer to make the treatment decision after hearing the doctor's opinion	9 (16.7)
I prefer to make the treatment decision together with the doctor	34 (63.0)
I prefer the doctor to make the treatment decision after talking to me	10 (18.5)
I prefer the doctor to make the decision on his/her own	1 (1.9)

*Note:* *N*– sample size, % – percentage.

**FIGURE 3:**
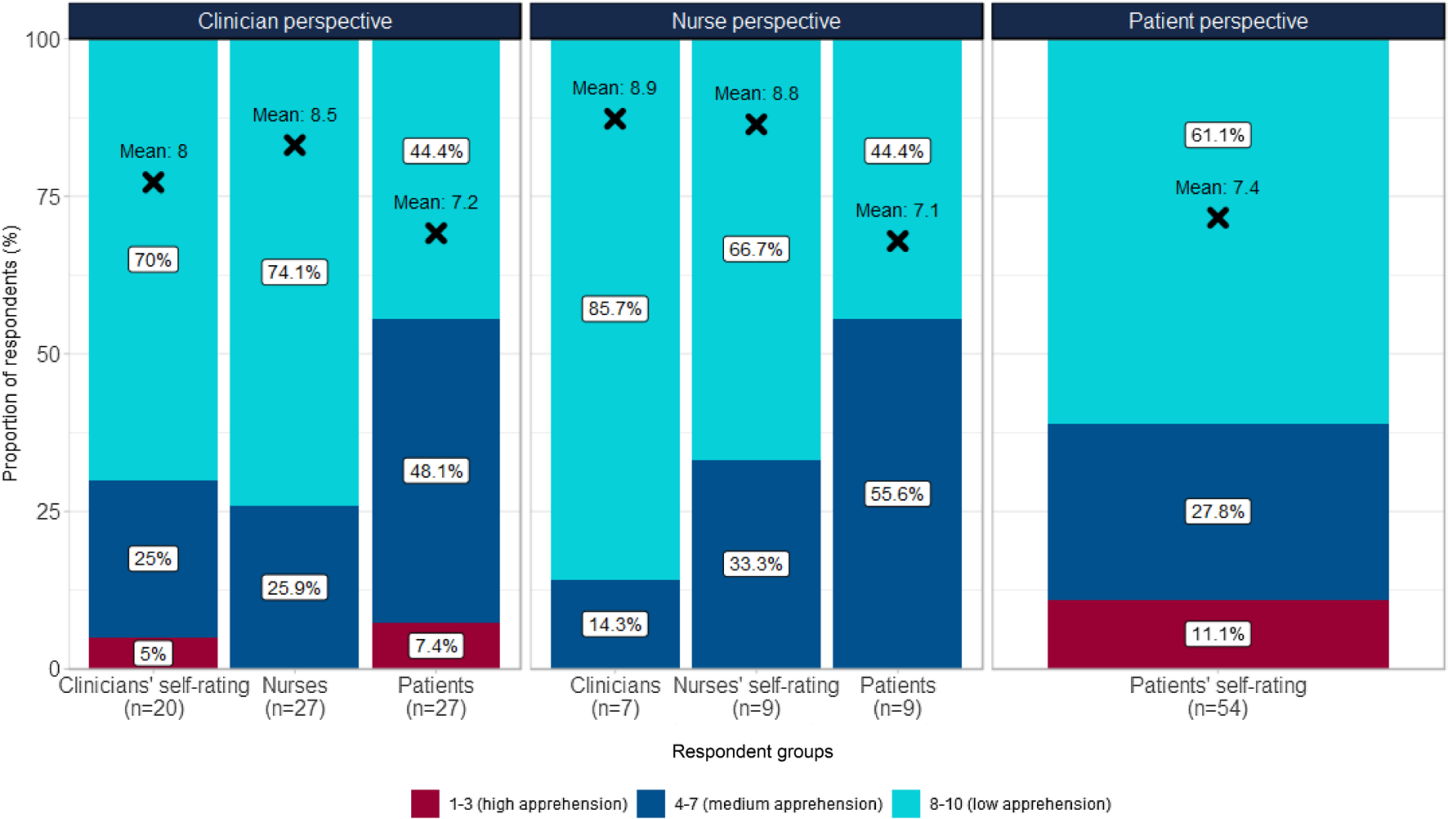
Comparison of patient, clinician and nurse perspectives for apprehension towards LA SSA injections.

Participants were also surveyed about their own apprehension towards LA SSA injections and their perception of others' apprehension (see Figure 3). 61.1% of patients with NETs indicated a low level of apprehension (ratings of 8–10), similar to 70.0% of clinicians and 66.7% of nurses reporting a low level of apprehension towards these injections.

Interestingly, 11.1% of patients with NETs rated their injection anxiety as high (ratings of 1–3), 27.8% as moderate (ratings of 4–7). On average, HCPs perceived patient apprehension scores similarly (patients reporting a mean score of 7.4 vs. 7.2 (from the clinicians' perspective) and 7.1 (from the nurses' perspective)); however, when considering levels of apprehension categorically, more HCPs perceived patients with NETs to have some or high levels of apprehension. HCPs considered only 44.4% of patients with NETs to have low levels of apprehension compared with 61.1% of patients. Clinicians perceived 48.1% of patients with NETs to be at least somewhat apprehensive (ratings of 4–7) about the injections and 7.4% to be highly apprehensive (ratings of 1–3); nurses perceived 55.6% of patients to be somewhat apprehensive (ratings of 4–7).

### 3.2 MMNL Model

The model estimates for the 54 patients with NETs, 27 clinicians and 9 nurses are reported in Table 5. The table exhibits the parameter coefficients, standard errors (SE) and associated *p* values. The distribution from which the random parameters were drawn was the constrained normal distribution. For the random parameter coefficients, the estimated mean value is reported.

**TABLE 5:** MMNL output parameters.

	Symbol	Estimate	CI LL (95%)	CI UL (95%)	Standard error	*T*‐ratio	*p*
Random parameters (means)							
Frequency							
Frequency^a^	FR_C	0.173	0.087	0.259	0.044	3.95	0.0001
Progression free survival							
PFS^a^	PFS_C	0.168	0.134	0.202	0.017	9.72	< 0.0000
Symptom control for diarrhoea & flushing							
Symptom control^a^	SYN_C	0.068	0.049	0.086	0.009	7.16	< 0.0000
Risk of GI side effects							
Risk of GI side effects^a^	GI_C	−0.073	−0.096	−0.050	0.012	−6.18	< 0.0000
Alternative‐specific constants (RC: Opt‐out)							
Deep subcutaneous injection	S1	−2.595	−3.660	−1.530	0.543	−4.78	< 0.0000
Deep intramuscular injection	I2	−2.772	−3.826	−1.717	0.538	−5.15	< 0.0000
Oral treatment	O3	−1.402	−2.281	−0.522	0.449	−3.12	0.0018
Heterogeneity in means of random parameters							
Progression free survival							
Progression free survival—patients^a^	PFS_:PAT	−0.048	−0.079	−0.018	0.016	−3.08	0.0021
Symptom control for diarrhoea and flushing							
Symptom control—patients^a^	SYN_:PAT	−0.022	−0.036	−0.008	0.007	−3.04	0.0024
Symptom control—metro^a^	SYN_:MET	−0.019	−0.034	−0.003	0.008	−2.37	0.0176
Risk of GI side effects							
Risk of GI side effects—metro^a^	GI_:MET	0.026	−0.001	0.053	0.014	1.88	0.0600
Random parameters (standard deviation)							
Frequency							
Frequency^a^	ZsFR_C	0.233	0.161	0.305	0.037	6.37	< 0.0000
Progression free survival							
PFS^a^	ZsPFS_C	0.100	0.081	0.119	0.010	10.25	< 0.0000
Symptom control for diarrhoea and flushing							
Symptom control^a^	ZsSYN_C	0.029	0.020	0.037	0.004	6.67	< 0.0000
Risk of GI side effects							
Risk of GI side effects^a^	ZsGI_C	0.028	0.009	0.048	0.010	2.91	0.0036
Alternative‐specific constants							
Deep subcutaneous injection	ZsS1	0.970	0.472	1.468	0.254	3.82	0.0001
Deep intramuscular injection	ZsI2	1.170	0.776	1.564	0.201	5.82	< 0.0000
Oral treatment	ZsO3	0.259	−0.427	0.945	0.350	0.74	0.4592

*Note:* Log‐likelihood: −837.30731; restricted log‐likelihood: −1497.19791; McFadden Pseudo *R* ^2^: 0.44; number of respondents: 90; number of choice observations: 1080.

Abbreviations: CI, confidence interval; LL, lower level; UL, upper level.

^a^ Continuous variable.

The treatment delivery attribute and patient support attribute for the deep subcutaneous (SC) (S1) and deep intramuscular (IM) (I2) injectable options, the treatment frequency for the oral option (O3) as well as the treatment administration attribute was not found to have a significant effect on choice and were removed from the final model.

The parameters around frequency of administration (FR_C), progression free survival (PFS_C), symptom control for diarrhoea and flushing (SYN_C) and the risk of GI side effects (GI_C) were specified as random parameters drawn from the constrained normal distribution. Differences in patient versus HCP preferences and metropolitan versus non‐metropolitan areas for these random parameters were tested within the model by trying to identify the source of heterogeneity in the means of the distribution. Results for this test are also displayed in Table 5.

The opt‐out was preferred over the three alternatives as shown by the negative and significant alternative‐specific constants. When only considering the three alternatives, oral was the most preferred treatment mode (−1.402), followed by the deep SC injection (−2.595) and the deep IM injection (−2.772). The observed difference between the deep SC and deep IM injection alternative constants was not statistically significant.

The positive parameter estimate for ‘frequency of administration’ (0.173) indicates an increase in preference with a lesser frequency of the injections. The positive parameter estimates for ‘PFS’ (0.168) and ‘symptom control for diarrhoea and flushing’ (0.068) indicate an increase in preference with a higher PFS or a higher degree of symptom control. The negative parameter estimate for ‘risk of GI side effects’ (−0.073) shows a decrease in preference with an increased risk of experiencing these side effects.

Regarding differences between patients with NETs and HCPs (please refer to heterogeneity in means of random parameters in Table 5), the attributes for ‘PFS’ and ‘symptom control for diarrhoea and flushing’ were again found to be significantly different between respondent segments, with both attributes more important to HCPs than to patients with NETs (−0.048 and −0.022, respectively). Additionally, this model also showed a significant difference between respondents from metropolitan vs. non‐metropolitan areas regarding ‘symptom control for diarrhoea and flushing’ (*p* = 0.0176). The negative parameter estimate of −0.019 for the metropolitan sample indicates that preference for this attribute was lower in the metropolitan sample compared to non‐metropolitan respondents. A lower significance was found for the ‘risk of GI side effects’ attribute, with the positive estimate of 0.026 for the metropolitan sample. This indicates a lower importance for this attribute in the metropolitan sample than the non‐metropolitan sample (i.e., the magnitude or strength of the parameter is not as strong).

### 3.3 Attribute Importance

In standard choice models, the relative attribute importance cannot be compared directly using attribute parameter size and significance. This is because the attributes represented by each parameter are presented on different scales. However, the model can be used to evaluate the importance of attributes relative to each attribute tested by comparing the utility derived from the lowest to the highest level for each attribute [31]. The relative importance of each attribute is calculated by finding the maximum difference between each attribute's level as a percentage of the total sum of all the maximum differences. The resulting change in utility for each attribute can be compared and used to calculate the relative attribute importance.

**FIGURE 4:**
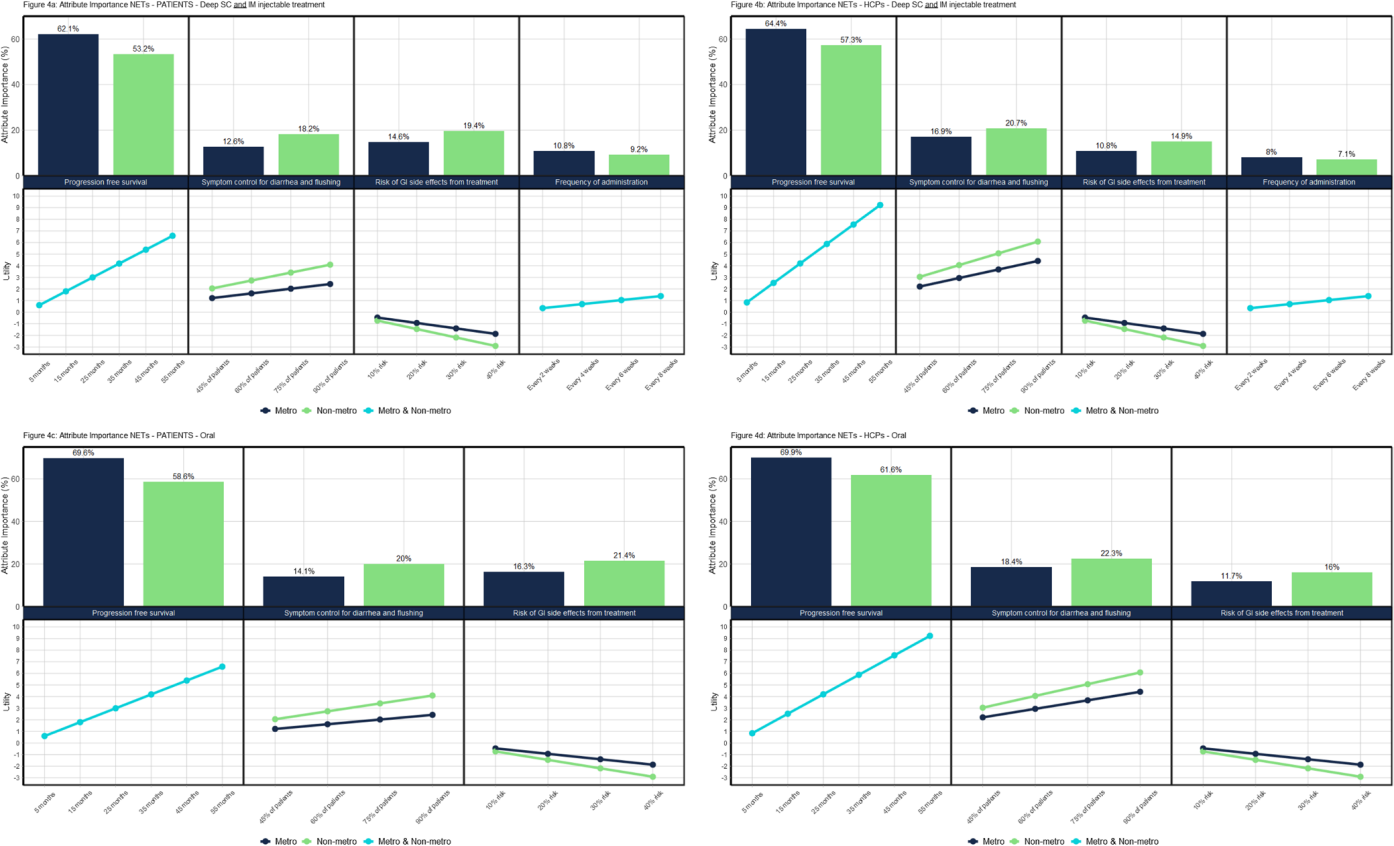
(a–d) Attribute importance by group.

Figure 4 represents the relative importance of each of the attributes used in the DCE. This figure shows the difference in utility for all attribute levels as well as the attribute importance based on this. Attributes with the biggest utility difference between the lowest and highest levels are the most important, as reflected by the attribute importance graphs.

Figure 4 illustrates attribute importance for two socio‐economic groups, metropolitan and non‐metropolitan, separately or combined. Please note that the relative attribute importance for the deep subcutaneous and intramuscular injections is identical; hence, the upper two graphs in Figure 4 represent both types of injections. Any attributes that were not found to be a significant predictor of preferences and therefore not included in the model have been excluded from Figure 4.

It can be seen in Figure 4 that ‘PFS’ is the most important attribute, followed by ‘symptom control for diarrhoea and flushing’, the ‘risk of GI side effects’ and ‘frequency of administration’ as shown by the incline of the respective lines. There are significant differences between respondents from metropolitan and non‐metropolitan areas for ‘symptom control for diarrhoea and flushing’ (*p* = 0.0176) and ‘risk of GI side effects’ (*p* = 0.0600), represented by the two different lines for each of these attributes in Figure 4. Both attributes are more important to respondents from non‐metropolitan than metropolitan areas as it can be seen by the steeper incline/decline of the green lines versus the dark blue lines.

For example, among patients evaluating oral treatment, progression free survival accounted for 69.6% of overall preference weight in metropolitan areas, compared with 58.6% in non‐metropolitan areas. Conversely, symptom control for diarrhoea and flushing was relatively more important to non‐metropolitan patients (20%) than to metropolitan patients (14.1%), and a similar pattern was observed for the risk of gastrointestinal side effects (21.4% vs. 16.3%). Among healthcare professionals, these trends persisted, with non‐metropolitan HCPs placing greater weight on symptom control and side effect risks than their metropolitan counterparts. These numerical differences illustrate how access to specialist care or proximity to treatment facilities may influence prioritisation of symptom management and side effect burden.

## 4 Discussion

This is the first study to examine treatment preferences and the relative feature importance for LA SSA treatments for NETs in an Australian context as well as the first study to compare preferences of patients with NETs and HCPs on this topic.

Overall, patients with NETs and HCPs treating NETs were aligned in their preferences for LA SSA treatments with ‘PFS’ as the most important attribute by far, followed by ‘symptom control for diarrhoea and flushing’, ‘risk of GI side effects’ and ‘frequency of administration’. The high importance of efficacy attributes such as ‘PFS’ is in line with findings from another DCE conducted with patients with NETs in Germany [32] where ‘overall survival’ was the key decision criterion. It is also in line with a review of DCE studies conducted in the oncology space, which shows that ‘overall survival’ and ‘PFS’ are commonly the most important attributes [33] for patients with NETs and HCPs, highlighting that efficacy measures provide the highest influence in treatment choice above all other attributes in NETs and in the oncology space in general.

Patients with NETs and HCPs were also aligned in preferring the oral alternative over the injectable, which reflects a general preference for oral modes of treatment administration over injection types of treatment. Despite this finding, the majority of patients with NETs and HCPs reported a comparatively low degree of apprehension around LA SSA injection (see Figure 3). This finding is supported by other studies that have also found a relatively low level of anxiety around SSA injections in patients with NETs: Darden et al. [34] reported that most patients with NETs in their study experienced either little (33.7%) or no (57.4%) anxiety immediately prior to their last SSA injection. Similarly, Ström et al. [35] reported that only 2%–11% of patients with NETs in their study showed moderate to severe anxiety before SSA injections. Darden et al. [34] also found that, among patients with NETs who did report some amount of anxiety, one of the most common reasons for this was the fact that they were not familiar with the nurse administering the injection. The fact that HCPs' perceptions of patient anxiety differed from patients' own perceptions in the current study, and that familiarity with the injector seems to be a key part of the injection experience, highlights the importance of having an open dialogue with patients and including them in their care decisions when treating individual patients with NETs.

HCPs valued clinical outcomes (‘PFS’ and ‘symptom control for diarrhoea and flushing’) higher than patients with NETs. The higher importance of clinical outcomes for HCPs than patients with NETs was also found in a systematic review of DCE studies conducted on oncology treatments by Collacot et al. [33]. Conversely, in the current study, attributes that may impact quality of life such as the ‘risk of GI side effects’ and ‘treatment frequency’ were relatively more important to patients with NETs than to HCPs, which reflects the value placed on quality‐of‐life measures for patients with NETs when considering treatment options. These differences among HCPs and patients with NETs indicate that there are varying perspectives on the ideal LA SSA treatment among patients and HCPs.

Among metropolitan and non‐metropolitan subgroups, differences were found in the degree of importance of the attributes ‘symptom control for diarrhoea and flushing’ and ‘risk of GI side effects’. These attributes related to symptoms were considered relatively more important for respondents in non‐metropolitan areas than in metropolitan areas. This potentially is influenced by the limited accessibility of medical services (including specialist care) in non‐metropolitan areas in Australia [36].

Patients with NETs in our study overwhelmingly expressed the desire to be involved in the treatment decision (98.1% in total), with 79.7% wanting to ‘make the treatment decision together with their doctor’ or ‘make the decision after hearing their doctor's opinion’. This, combined with the fact that HCPs perceive patients' injection apprehension to be more pronounced than reported by the patients themselves, highlights the need for clinicians to actively educate patients on their treatment options and include their patients in their treatment decisions when prescribing an LA SSA for NETs.

Overall, the findings demonstrate that while clinical treatment attributes are important, when making treatment decisions, patients with NETs and HCPs also consider attributes that impact quality of life such as symptom control, adverse events and frequency of treatment. Furthermore, while the relative ranking of attributes was consistent among all groups analysed, the fact that HCPs value clinical outcomes relatively higher than patients with NETs, who value quality of life measures slightly higher, indicates some variance in preference. Similar differences were seen in the metropolitan and non‐metropolitan subgroups, with symptom control having a higher degree of importance for those in non‐metropolitan regions. These results highlight that differences exist between preferences among patients with NETs and HCPs and may be further influenced by location. This provides important information beyond what can be examined in clinical trials and has practical implications for patients with NETs and their care team when discussing treatment choices. Previous research has shown that patient‐clinician alignment in treatment preferences can lead to improved treatment adherence and subsequently improved treatment outcomes [15, 16]. Given the long duration of use of LA SSAs in the treatment for NETs, it is anticipated that understanding the similarities and differences in treatment preferences between patients with NETs and HCPs will serve in supporting treatment choice and highlight the importance of including patients in the treatment decision.

Our findings highlight the importance of patient and clinician preferences in treatment decision‐making. These preferences should be considered in light of emerging mechanistic insights, evidence on long‐term outcomes, evolving therapeutic strategies and side‐effect management. Recent innovations, such as artificial intelligence (AI) based approaches that incorporate patient queries into clinical decision‐making for neuroendocrine neoplasms, further demonstrate the value of patient‐centred tools [37]. Integrating patient‐centred evidence with clinical data can strengthen shared decision‐making and support tailored care strategies for people living with NETs. Results also highlight the need for improved communication between patients and healthcare professionals. Incorporating structured shared decision‐making frameworks may help ensure that patient experiences are more accurately understood and addressed in treatment planning [38, 39, 40].

In terms of limitations, the sample size, in particular for HCPs, was comparatively small. The achieved sample size also meant that the options for sub‐population analysis were limited, which may have left other factors uncovered. This means the findings should be interpreted with caution in terms of generalizability; however, they still offer valuable insights into treatment preferences in this under‐researched area. Furthermore, respondents were asked to evaluate certain hypothetical treatment scenarios, which may not necessarily reflect the choices they would make in a real setting. Lastly, the dominance of the PFS attribute in decision‐making may have impacted non‐significant attributes that could possibly have come out as significant if PFS had not been included (although this would have been an unrealistic scenario to test).

At the time of research design and data collection (2021–2022), our study focused on attributes of currently available LA SSAs. Future research could explore how preferences shift in response to attributes of newer treatment options and whether they provide clinical or practical advantages.

## 5 Conclusions

Overall, the study shows that clinical as well as nonclinical treatment attributes are important to patients with NETs and HCPs treating these patients to a varying degree. While there is a high degree of alignment between patients with NETs and HCPs in the treatment attributes they value (‘PFS’ and ‘symptom control for diarrhoea and flushing’ were found to be the most important), the results also show some varying perspectives on LA SSA treatments with regard to the magnitude of importance placed on treatment attributes by different subgroups. These findings have practical implications around understanding the needs and wants of patients with NETs and the HCPs that treat them. They highlight the value of including patients in prescribing decisions to optimise treatment choice and ultimately treatment outcomes.

## Author Contributions

**Simon J. Fifer:** conceptualization (lead); methodology (lead); formal analysis (lead); writing – review and editing (equal). **Karen Winkler:** conceptualization (supporting); methodology (supporting); writing – review and editing (equal). **Meredith Cummins:** conceptualization (supporting); methodology (supporting); writing – review and editing (equal). All authors: formal analysis (supporting); writing – review and editing (equal); approval of final manuscript.

## Ethics Statement

This study was approved by the Bellberry Limited Human Research Ethics Committee, Australia (protocol number 2020‐10‐1038).

## Consent

All participants provided informed written consent prior to participating in the study.

## Conflicts of Interest

S.J.F. is an employee of CaPPRe. K.W. is a former employee of CaPPRe and was working as a contractor for CaPPRe during the time of this research. CaPPRe has consulted to Abbvie, Amgen, AstraZeneca, BMS, CSL Behring, Edwards, Gilead, GSK, Ipsen, Janssen, Medtronic, Novartis, Novo Nordisk, Roche, Seqirus and Vertex, outside of the submitted work. M.C. is an employee of NeuroEndocrine Cancer Australia. H.C. was affiliated with Ipsen Australia at the time of the study.

## Supporting information


**Data S1:** cam471323‐sup‐0001‐Supinfo.docx.

## Data Availability

Restrictions apply to the availability of these data since the data underlying this publication were provided by CaPPRe under contract to Ipsen.
