# Supplementary material for: Attribute Preferences for Somatostatin Analogues in Neuroendocrine Tumours (NETs) Among Patients, Clinicians and Nurses in Australia
Source: Cancer Med. 2025 Nov 12;14(21):e71323. doi: 10.1002/cam4.71323 (PMC12606003; doi:10.1002/cam4.71323)
Supplement: Supplementary file 1 — Data S1: cam471323‐sup‐0001‐Supinfo.docx. [file CAM4-14-e71323-s001.docx]

**Supplementary information: Plain language summary**

Neuroendocrine tumours (NETs) are a type of cancer that develop from cells in the hormone-producing and nervous systems. This Australian study investigated what is important to patients with NETs, their doctors and nurses when choosing a treatment. The study focused on long-acting somatostatin analogues (LA SSAs), a medication often used as the first treatment option for patients with NETs.

An online survey was completed by 54 patients, 27 doctors, and 9 nurses. Participants were presented with multiple scenarios and in each, were asked to choose between three possible treatment options (injections under the skin, injections into the muscle, or an oral tablet) or to select no treatment. Each treatment option was described using several different features such as how long the treatment stops the tumours from growing, how often the treatment needs to be taken, symptom control (e.g., diarrhea and flushing), and risk of side effects in the stomach and intestines.

Most people preferred the oral treatment to injections. The most important factor for all participant groups (patients, doctors and nurses) was how long the treatment stopped the tumours from growing, followed by how well it controlled symptoms and the risk of side effects in the stomach and intestines. However, doctors and nurses placed greater importance on how long the treatment worked and how well it controlled symptoms than patients did, whereas patients cared more about features that impact quality of life, such as side effects in the stomach and intestines, and how often the treatment needs to be taken. In addition, patients, doctors and nurses living outside of larger cities considered controlling symptoms and the risk of side effects to be more important than those living in larger cities. These findings show it is important for patients, doctors and nurses to have open discussions about options when choosing a medicine for the treatment of NETs.
